# Timeliness of contact tracing among flight passengers for influenza A/H1N1 2009

**DOI:** 10.1186/1471-2334-11-355

**Published:** 2011-12-28

**Authors:** Corien M Swaan, Rolf Appels, Mirjam EE Kretzschmar, Jim E van Steenbergen

**Affiliations:** 1 Preparedness and Response Unit, Centre for Infectious Disease Control, National Institute for Public Health and the Environment (RIVM), A. van Leeuwenhoeklaan 9, 3721 MA Bilthoven, the Netherlands; 2 Municipal Health Service, GGD Kennemerland, Spaarnepoort 5, 2134 TM Hoofddorp, the Netherlands; 3 Julius Centre for Health Sciences & Primary Care, University Medical Centre Utrecht, Heidelberglaan 100, 3584 CX Utrecht, the Netherlands; 4 Epidemiology and Surveillance Unit; Centre for Infectious Disease Control, National Institute for Public Health and the Environment (RIVM), A. van Leeuwenhoeklaan 9, 3721 MA Bilthoven, the Netherlands

## Abstract

**Background:**

During the initial containment phase of influenza A/H1N1 2009, close contacts of cases were traced to provide antiviral prophylaxis within 48 h after exposure and to alert them on signs of disease for early diagnosis and treatment. Passengers seated on the same row, two rows in front or behind a patient infectious for influenza, during a flight of ≥ 4 h were considered close contacts. This study evaluates the timeliness of flight-contact tracing (CT) as performed following national and international CT requests addressed to the Center of Infectious Disease Control (CIb/RIVM), and implemented by the Municipal Health Services of Schiphol Airport.

**Methods:**

Elapsed days between date of flight arrival and the date passenger lists became available (contact details identified - CI) was used as proxy for timeliness of CT. In a retrospective study, dates of flight arrival, onset of illness, laboratory diagnosis, CT request and identification of contacts details through passenger lists, following CT requests to the RIVM for flights landed at Schiphol Airport were collected and analyzed.

**Results:**

24 requests for CT were identified. Three of these were declined as over 4 days had elapsed since flight arrival. In 17 out of 21 requests, contact details were obtained within 7 days after arrival (81%). The average delay between arrival and CI was 3,9 days (range 2-7), mainly caused by delay in diagnosis of the index patient after arrival (2,6 days). In four flights (19%), contacts were not identified or only after > 7 days. CI involving Dutch airlines was faster than non-Dutch airlines (*P *< 0,05). Passenger locator cards did not improve timeliness of CI. In only three flights contact details were identified within 2 days after arrival.

**Conclusion:**

CT for influenza A/H1N1 2009 among flight passengers was not successful for timely provision of prophylaxis. CT had little additional value for alerting passengers for disease symptoms, as this information already was provided during and after the flight. Public health authorities should take into account patient delays in seeking medical advise and laboratory confirmation in relation to maximum time to provide postexposure prophylaxis when deciding to install contact tracing measures. International standardization of CT guidelines is recommended.

## Background

Aircrafts can function as transport vehicle for patients infected with influenza, leading to introduction of a new virus strain to non-endemic areas [1,2]. Although the risk is small, passengers might be infected by a contagious patient during the flight [3-6], as well as during public transport [7]. Transmission during the flight increases the possibility of further transmission in the area of destination. For these reasons, during the initial phase of the influenza A/H1N1 2009 pandemic, many countries initiated contact tracing among flight passengers of flights where contagious patients with laboratory confirmed influenza A/H1N1 2009 were notified.

A risk assessment guideline for infectious diseases transmitted on aircrafts has been developed by the European Centre for Disease Prevention and Control (ECDC) [8], which includes influenza. Literature study revealed on-board transmission in flights with a duration of less than 8 h. The majority of infected contacts during these flights were seated on the same row, or one or two rows in front of behind the index [9-12]. Contacts up to 8 and 10 rows distance from the index were infected in one study [10]. As these contacts also had personal contact with the index during the flight, transmission across a distance of so many rows is not proven. The guideline concludes that it is difficult to design a single contact tracing algorithm for influenza. Due to the short incubation period of influenza, it is almost impossible to provide contacts with postexposure prophylaxis (PEP) within the time that it is most effective, which is 48 h after exposure [13]. Therefore, the main aim of contact tracing might be to interrupt the chain of transmission by alerting contacts for early diagnosis and treatment. Although the World Health Organization (WHO) developed technical advice for case management of influenza A/H1N1 2009 in air transport during the pandemic [14], no international standardized protocol for contact tracing for this pathogen was available.

In line with the ECDC guideline [8] and the Dutch guideline for 'Incidental introduction of a new influenza strain' [15], in the Netherlands close contacts of a patient with laboratory confirmed pandemic influenza were identified. In case the index had been contagious during a flight with a duration of ≥ 4 h, passengers and cabin crew were to be informed on signs and symptoms of the disease and to seek medical care in case they would occur. In addition, close contacts, defined as passengers seated on the same row, two rows in front and two rows behind the index case, as well as the cabin crew working in this compartment, were traced by public health authorities to provide a 10 day prophylactic course of oseltamivir as soon as possible (preferably within 48 h after exposure).

Schiphol Airport is the only airport in the Netherlands where trans-Atlantic flights arrive. Its Municipal Health Services (MHS, GGD Kennemerland) and the Center for Infectious disease Control (CIb-RIVM) frequently experienced that, despite all efforts, the time period elapsing from exposure to administration of the first oseltamivir dose exceeded the required 48 h. Acquiring contact details from airlines was time consuming, and contact details on passenger lists were often minimal, so that contacts were difficult to trace. In this study, we assess the time delay in contact tracing of flight passengers for influenza A/H1N1 2009 as performed in the Netherlands during the initial phase of the pandemic. Our data show that despite all efforts the effectiveness of this control measure in daily practice is minimal.

## Methods

From April 29th until June 22nd 2009, contact tracing among flight passengers in the Netherlands was indicated for laboratory confirmed influenza A/H1N1 2009 cases, who traveled on a flight for 4 h or longer while being contagious, defined as 1 day before, until 7 days after disease onset. These criteria were installed by the CIb, which also functions as National focal point (NFP).

**Figure 1:**
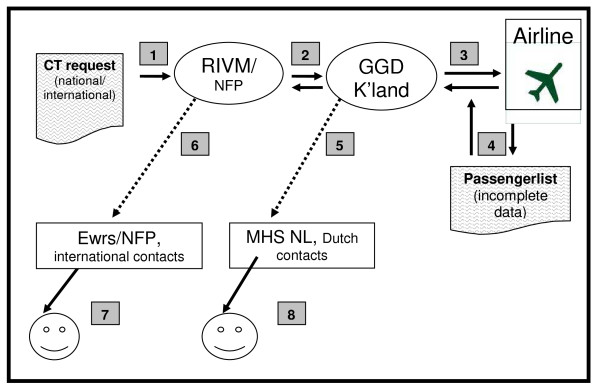
**Routing of contact tracing for close passenger flight contacts during the influenza A/H1N1 2009 pandemic**. A request for contact tracing (CT) reaches the NFP of the RIVM (1). After checking the indication, the request is forwarded to the MHS/GGD Kennemerland (2) who will submit the request at the appropriate airline (3). The airline collects contact details of the passengers and returns this to the GGD (4). Dutch contacts will be informed through the MHS of their region (5, 8), international contacts list are returned to the NFP/RIVM. The NFP requests the concerned public health authorities through the NFP or EWRS contact points to trace the contacts (6, 7).

The procedure for contact tracing is complex, see Figure 1. Requests for contact tracing to the CIb for Dutch index patients originate from any Dutch MHS which identifies a patient who traveled by plane while being contagious for an infectious disease which requires contact tracing. Other nation's health authorities will make a request to the CIb in case they diagnosed a patient which arrived at Schiphol airport for transit while being infectious. Requests for CT in the last group are submitted to the National Focal Point (NFP) or through the Early Warning and Response system of the EU (EWRS). The CIb verifies laboratory confirmation, and the indication for contact tracing regarding flight duration. The MHS of the airport where the specific flight landed coordinates contact tracing for flight passengers. In case of Schiphol, MHS Kennemerland approaches the involved airline company requesting the passenger list. The airline provides passenger lists with at least passenger names, seat numbers and booking or contact details. MHS Kennemerland then completes contact details through booking offices or using other search methods. Close contacts living in the Netherlands are traced by the respective Dutch MHS's. For tracing foreign contacts, the CIb sends a notification with contact details to the NFP of the country of final destination, or through the EWRS system for EU countries.

During the pandemic, CT requests were turned down if more than 4 days had elapsed after flight arrival, as contact tracing was not considered to have additional value. During the study period, passenger locator cards (PLC) only were used on direct flights from Mexico during the initial phase of the pandemic. These flights were all run by Dutch airlines.

**Figure 2:**
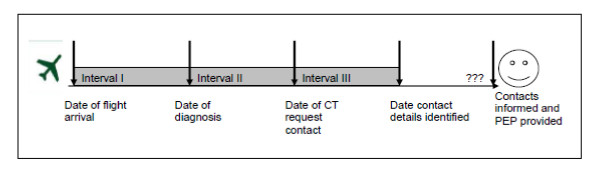
**Delay intervals in contact tracing amongst flight passengers**. Interval I, II and III are defined as serial intervals between the dates of flight arrival, date of diagnoses, date of contact tracing request and date of contact details identified respectively.

For each contact investigation performed in the period April 29th until June 22nd 2009, the following data were collected: flight arrival date, first day of illness of index patient, date of laboratory diagnosis, date of contact tracing request and the date passenger lists were obtained and contact details were completed ('contacts details identified'). From these data, time intervals (in days) between flight arrival and date of diagnosis (interval I), between diagnosis and request dates (interval II) and between request and contact details identified dates (interval III) were calculated, see Figure 2. Date of actual contact tracing and oseltamivir administration was not available in this study, but is inherently always hours if not days later. As the airline company traces contacts amongst crewmembers, these are not included in this study.

Data were analyzed using SPSS software (version 18, USA). The influence of availability of PLC's on timeliness and the origin of the airline company (Dutch or non-Dutch) were statistically analyzed.

## Results

In the period April 29th until June 22nd 2009, 24 indications for CT were identified. Three international requests concerning CT for influenza patients diagnosed outside the Netherlands were declined as already more than 4 days had elapsed since flight arrival. In 17 out of the 21 remaining contact investigations, passenger lists with contact details were obtained within 7 days after arrival (81%), see Table 1. In total contact details of 451 close contacts were identified, of which 199 contacts lived in the Netherlands, and 252 contacts abroad. The average number of close contacts per flight was 27 (range: 8-44). In four contact investigations (19%), contact details were not obtained, or provided later than 7 days after flight arrival and CT was stopped. These CT were all related to non-Dutch airlines, and total delay was stated 8 days for further data processing. Of the 21 requests, the total delay between request and contact detail identification was longer for non-Dutch airlines (mean 6,3 SD 2,7) compared with Dutch airlines (mean 4.1 days, SD 1,5)(1-sided Mann-Whitney test, *p *= 0,033).

**Table 1:** Overview of 17 contact investigations (n.a.: information on these dates was not available)

Nr	Flight arrival date Schiphol	Dutch (DA)/foreign airline (FA)	Origin	PLCª used	First date of illness	Date of diagnosis	Date request CT	Date contacts identified	Interval I: flight arrival - diagnosis	Interval II: diagnosis - request CT	Interval III: request CT - contact identified	Total Delay (I + II + III)
1	27-4-09	DA	Cancun	No	27-4	29-4	29-4	30-4	2	0	1	3

2	30-4-09	DA	Cancun	No	30-4	6-5	7-5	7-5	6	1*	0	7

3	6-5-09	DA	Mex city	Yes	4-5	7-5	8-5	8-5	1	1	0	2

4	6-5-09	DA	Mex city	Yes	6-5	11-5	12-5	12-5	5	1**	0	6

5	14-5-09	FA	Detroit	No	12-5	n.a.	16-5	16-5	***		0	2

6	21-5-09	DA	NewYork	No	22-5	n.a.	25-5	26-5	***		1	5

7	29-5-09	DA	Houston	No	29-5	n.a.	2-6	2-6	***		0	4

8	29-5-09	DA	Houston	No	n.a.	3-6	4-6	4-6	5	1**	0	6

9	2-6-09	DA	NewYork	No	2-6	3-6	4-6	5-6	1	1	1	3

10	8-6-09	DA	Toronto	No	2-6	n.a.	10-6	11-6	***		1	3

11	13-6-09	DA	NewYork	No	13-6	15-6	16-6	16-6	2	1	0	3

12	18-6-09	DA	Cancun	Yes	13-6	19-6	19-6	20-6	1	0	1	2

13	14-6-09	DA	Miami	No	13-6	16-6	17-6	18-6	2	1	1	4

14	9-6-09	FA	Boston	No	7-6	11-6	12-6	13-6	2	1	1	4

15	14-6-09	DA	Cancun	Yes	11-6	16-6	17-6	17-6	2	1	0	3

16	14-6-09	DA	Cancun	Yes	14-6	17-6	18-6	19-6	3	1	1	5

17	18-6-09	DA	Miami	No	18-6	20-6	20-6	23-6	2	1	2	5

**Mean (95%CI)**									**2,6** **(1,6-3,6)**	**0,8** **(0,6-1,1)**	**0,6** **(0,3-0,9)**	**3,9** **(3,2-4,7)**

*:. In the beginning of the pandemic one request for contact tracing was accepted after 7 days

**: these late CT requests were accepted as the passenger lists of the concerned flights already were available from earlier contact investigations

***: date of diagnosis not known

ª: Passenger Locator Card

**Figure 3:**
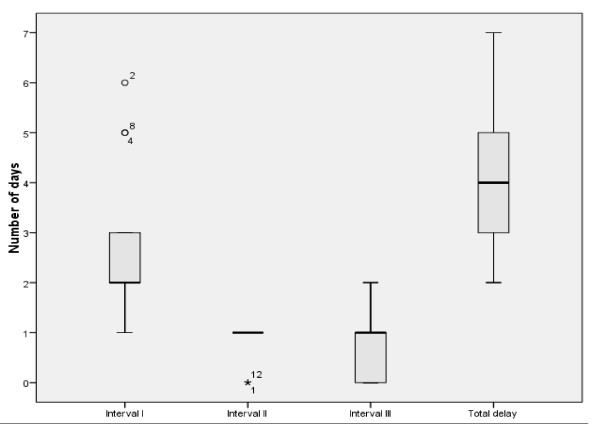
**Box plot distribution of serial intervals**. The boxplox show Intervals I (delay flight arrival and date of diagnosis), interval II (delay date of diagnosis and CT request) and interval III (date of CT request and contact details identified), representing median, 25%-75% Quartile (IQR, in boxes), values between 1,5 IQR (lines) and outliers (* and ° with flight numbers).

For the 17 completed contact investigations, interval I was the largest interval in the contact tracing procedure (mean 2,6 days, range 1-6, 95% CI 1,6-3,6, n = 13). The other intervals II and III were shorter, with a mean of 0,8 days and 0,6 days respectively, see Table 1. Figure 3 shows the medians of the described intervals. Since 15/17 index cases were already ill before, or during the day of arrival of the flight, the delay in interval I is mainly caused by delay in seeking medical advice and diagnostic procedure itself. After acceptance of the request for CT by the CIb, GGD Kennemerland needed on average 0,6 days (range 0-2, 95% CI 0,3-0,9 days) to collect the passenger list from the airlines and complete contact details (interval III). The total delay between flight arrival and identification of contact details was on average 3,9 days (range 2-7 days, 95% confidence interval 3,2-4,7 days), see Table 1. In only 3 out of 17 contact investigations (18%), contacts were identified within 2 days after arrival. In 2 out of these 3 contact investigations, PLC's were available. Interval III of the 5 CT with PLC's available was shorter (0,4 days, SD 0,5) than for 12 CT's without PLC (0,7 days, SD 0,7), this was not significant however (p: 0,25). Overall delay in CT with PLC's also was shorter (mean 3,6, SD 1,8), but not significant, when compared to CT without PLC's (mean 4,1, SD 1,4) (p:0,25).

## Discussion and Conclusions

In this study we evaluated the timeliness of contact tracing (CT) of flight contacts in daily practice. We conclude that the prevailing policy to provide close contacts antiviral PEP during the early phase of the influenza pandemic is very difficult to implement effectively and therefore has little effect to control disease spread. Active case finding through contact tracing of exposed persons is an important procedure during the containment phase of an emerging communicable disease. However, our data show that, even in a small-industrialized country with modern communication tools, tracing of flight contacts exceeds the required maximum of 48 h after exposure.

For influenza, close contacts of contagious index cases are entitled to receive antiviral PEP within 48 h after exposure to prevent them from becoming ill and further spreading of the disease. Starting oseltamivir within 48 h does not prevent disease but shortens the disease period, mitigates symptoms and might decrease further transmission. Awareness among contacts to seek medical evaluation when influenza-like (ILI) symptoms occur, for both proper antiviral treatment and (home-) isolation advice, reduces further spreading. As influenza has a relative short latent period, for influenza A(H1N1)/2009 varying between 0,7-3,1 days [16,17], contacts ideally should be informed within 1 day. Oseltamivir postexposure prophylaxis for this pandemic strain is reported to be effective even when administrated more than 48 h after exposure in household settings [18], however, delays in administration are not specified. We cannot exclude the possibility that in our study, even delayed administration of oseltamivir prophylaxis may have prevented some people from becoming ill, although we anticipate the effectiveness of the intervention overall to be less in this setting than in households.

Our study among 17 contact investigations showed an average total delay of 3,9 days between flight arrival and identification of contacts by passenger list, which is too late for effective PEP, and late for alerting on first symptoms of disease. Only in three contact investigations (18%), contact details were obtained within 48 h. However, after identification of passenger details, health authorities need time to actually trace the contact and administer PEP. It is highly unlikely that this was achieved within the same 48 h. We therefore conclude that contact investigation for provision of PEP as conducted here was ineffective.

Regarding the awareness of ILI symptoms, Schiphol Airport handed all passengers on flights arriving from Mexico information leaflets on influenza A/H1N1 2009 with information on early symptoms and requesting them to seek medical advice in case of fever and respiratory symptoms such as coughing. Posters with this information were placed in passenger halls, to inform passengers arriving indirectly from Mexico via transit through other airports, or arriving from non-endemic areas with higher transmission (e.g. USA). As contact details were identified on average 3.9 days after exposure, however not contacted yet, we conclude that CT did not have additional value for timely achievement of increased awareness.

It is not a new finding that contact tracing of flight passengers is a time-consuming procedure [8]. In one study among flight passengers during the pandemic in 2009, 52% (53/95) of the contacts were reached within 72 h [5]. In a measles contact investigation, 75% (202/275) of responding passengers were contacted within 72 h. In this study however, the diagnosis of measles was already suspected during the flight, and laboratory confirmation was initiated immediately after landing [19]. It also helped that many contacts were tourists staying at the same hotels, which facilitated tracing them.

Our study shows that the longest delay before identification of contact details for an influenza index case is caused by the time between arrival and laboratory diagnosis (interval I, 2,6 days). This delay is a result of patients delay in seeking medical care, and doctor's delay, including laboratory confirmation. For influenza, the indicated laboratory test was Polymerase Chain Reaction, which takes several hours to obtain the result and in the beginning of the pandemic, the PCR test was not yet available in many laboratories.

Patients delay was considerable however. It even took the seven passengers with date of onset before the flight, and therefore symptomatic during the flight, 1 to 2 days after arrival before laboratory confirmation was made. Also, none of the airline reported that these patients already were identified during the flight, nor that infection control measures were taken. For the indexes that became ill on the day of arrival, delay until laboratory confirmation still lasted 3 days (range 1-6 days). A pre-pandemic study by Sharangpani et al. among flight passengers showed that they are more willing to seek physicians care in case they developed flu-like symptoms when the perceived the pandemic as serious [20]. Leggat et al. demonstrated during the pandemic that only a minority (35,5%) of Australian citizens would cancel their air travel in case of cough and fever lasting more than 1 day. This was higher among persons who were more concerned about the pandemic [21]. In the Netherlands, the perceived severity of the disease decreased significant during this study period [22]. We expect that the delay until laboratory diagnoses in this study considerably is affected by patients delay seeking medical care, which might be better in diseases experienced as more threatening.

Collecting passenger details from foreign airlines also caused considerable delay because of differences in time zones and the need to convince the concerned airline companies about the urgency to collect and hand-over passenger lists with contact details. Sometimes official request letters were necessary for legal reasons to release personal contact details. Dutch companies were easier to convince by Dutch health authorities to hand over passenger details. Our data show that contact details that were identified too late or not at all, indeed more often originated from non-Dutch than from Dutch airline companies. An internationally standardized contact tracing protocol, communicated with the International Civil Aviation Organization (ICAO) and International Air Transport Association (IATA), would facilitate the timeliness, and therefore effectiveness of contact tracing.

Although one might expect differently, timeliness of CT for flights where PLC's were available, was not better than CT for flights without PLC. However, PLC's reduces the effort, in terms of staff support for airline companies and the municipal health service to collect useful passenger information considerably. PLC's were only used by Dutch airlines, who already were able to provide passenger lists relatively quickly. This also explains the limited attributed shortening in timeliness. Contact details on PLC's might be more accurate to trace the passenger than details provided by the passenger list or booking station. This is further investigated.

This study has several limitations. As available data were recorded in days, and not in hours, it was not possible to determine the time intervals more precisely. As this was both with first and last date of the intervals, we expect no negative or positive bias. Secondly, the arrival date was used for date of exposure, while the actual exposure might have already taken place the day before at departure of the flight. This would imply an increase in delay and decrease the effectiveness of contact tracing. Also, we have no data if, and when contacts were actually reached and oseltamivir was administered. Since several steps were still required to reach the contacts after they were identified through passenger lists, this only would have lead to further delay in administrating prophylaxis. Further investigation into the timeliness of administration of prophylaxis among these contacts is initiated, to have insight in the delay of this last interval to facilitate future decisions on the effectiveness and necessity of contact tracing among flight passengers. Lastly, this study includes CT initiated at only one airport. CT procedures might be different at airports in other countries, which influences interval III. As this is not causing the main delay, we do not expect that in other countries CT would be much faster.

We conclude that tracing close contacts among flight passengers during the initial phase of pandemic A/H1N1 2009 was not effective, as timely provision of PEP could not be achieved in most cases. Most contacts came from an endemic area (Mexico) or areas with well known increased transmission during the first 2 months of the pandemic. The additional risk for those travelers of being a close contact during a long haul flight is small (3,5%) [5]. Furthermore, airline companies and/or Schiphol airport already provided contacts with information on the disease and its symptoms by. The benefit to inform them of the fact that they were contacts of a laboratory confirmed case did not justify the extra effort health authorities invested in contact tracing, especially during a period where public health officials, airports and airline companies were absorbed by efforts of other pandemic related control measures.

In hindsight, the limited burden of disease of influenza A/H1N1 2009 did not justify contact tracing efforts. The main reason for flight contact tracing is raising alertness for possible exposure to uncommon infectious diseases, enabling early recognition and treatment of the disease and timely installation of control measures (e.g. SARS and viral hemorrhagic fevers). For some diseases, PEP is indicated as well. The risk assessment upon which the decision to install contact tracing is based should incorporate - apart from an evaluation of the severity and rarity of disease - an assessment of the required timeliness of effective control measures [23]. The expected time for laboratory confirmation of index cases and identification and tracing of contacts should be related to the maximum period during which quarantine, PEP or other control measures are effective in order to decide on the benefit of this time consuming procedure. Lastly, also cabin crew should be aware of their role of signaling infectious patients. In consultation with medical professionals, direct control measures can be installed, as well as medical evaluation after landing.

## Conflict of interests

The authors declare that they have no competing interests.

## Authors' contributions

CS and RA designed the study and collected the data. MK advised on the data management and presentation of the results. JS, CS and RA interpreted the data. JS critically revised the manuscript. CS wrote the manuscript and all authors commented on drafts and approved the final version. All authors read and approved the final manuscript.

## Pre-publication history

The pre-publication history for this paper can be accessed here:

http://www.biomedcentral.com/1471-2334/11/355/prepub
